# Introductory Lecture to the Course of Instruction in the Medical Institution of Geneva College

**Published:** 1842-12-24

**Authors:** 


*Introductory Lecture to the Course of Instruction in the Medical Institution of Geneva College*, (*upon the occasion of opening their New Building*, 4th Oct., 1842.) By Thomas Spencer, M. D., Dean, Professor of the Institutes and Practice of Medicine. Geneva, New York : 1842. 8vo. pp. 19.

This is a rapid and cleverly written sketch of the history of medicine, and, in addition, contains the usual quota of sound advice, pleasantly conveyed. Our limits prevent us from indulging in several extracts which we had marked.

